# Red blood cell distribution width-to-albumin ratio and postoperative pleural effusion risk after gastrectomy for esophagogastric junction adenocarcinoma: a retrospective study

**DOI:** 10.3389/fonc.2026.1746267

**Published:** 2026-03-11

**Authors:** Zhongqiang Zheng, Guanglin Qiu, Kui Yang, Yingmu Tong, Lindi Cai, Min Wang, Xinhua Liao, Xiangming Che, Lin Fan

**Affiliations:** 1 Department of General Surgery, The First Affiliated Hospital of Xi’an Jiaotong University, Xi’an, Shaanxi, China; 2 Endocrinology Department, Xi’an Beihuan Hospital, Xi’an, Shaanxi, China

**Keywords:** adenocarcinoma of the esophagogastric junction, biomarker, gastrectomy, postoperative pleural effusion, red blood cell distribution width-to-albumin ratio

## Abstract

**Background:**

Postoperative pleural effusions (PPE) are common complications following gastrectomy, particularly in patients diagnosed with Siewert type II/III esophagogastric cancer. The red blood cell distribution width-to-albumin ratio (RAR) is a marker indicative of systemic inflammation and nutritional status. This study investigates the association between preoperative RAR and the incidence of PPE in patients with Siewert type II/III adenocarcinoma of the esophagogastric junction (AEG) who have undergone gastrectomy.

**Methods:**

A retrospective analysis was performed on data from 299 patients with Siewert type II/III AEG who underwent gastrectomy between January 2020 and December 2024. Logistic regression analysis was utilized to assess the relationship between RAR and PPE, while the receiver operating characteristic curve was employed to evaluate the predictive capability of RAR for PPE risk, with results expressed as the area under the curve (AUC).

**Results:**

PPE was diagnosed in 85 patients, representing 28.43% of the study cohort. After adjusting for relevant covariates, a positive association between the RAR and PPE following gastrectomy was observed (odds ratio [OR] = 1.69, 95% confidence interval [CI]: 1.22–2.34, P = 0.002). The incidence of PPE demonstrated a progressive increase across ascending RAR quartiles, with patients in the highest quartile experiencing a significantly increased risk of PPE compared to those in the lowest quartile (OR = 4.15, 95% CI: 1.28–13.45, P = 0.02). Furthermore, RAR exhibited a strong predictive capacity for PPE risk, as indicated by an AUC of 0.796. Subgroup analysis further confirmed a significant positive association between RAR and PPE. Stratified analysis revealed significant interactions between hemoglobin levels (P = 0.04) and the effect of RAR on PPE.

**Conclusion:**

An elevated RAR is independently associated with an increased risk of PPE, suggesting its potential utility as a pragmatic, clinically feasible supplementary index for patients with Siewert type II/III AEG prior to undergoing gastrectomy. However, due to the study’s single-center, retrospective design and absence of external validation, further research with external cohorts is needed before RAR can be considered for widespread clinical implementation.

## 1 Introduction

Gastric cancer (GC) continues to be one of the most prevalent malignancies globally, presenting significant public health challenges (1, 2). Despite recent declines in both its incidence and mortality rates, GC remains a leading cause of cancer-related mortality (1, 3). For resectable GC, particularly when diagnosed at an early stage, surgery remains the sole potentially curative treatment option (4, 5). The standard treatment protocol involves radical resection with lymph node dissection, with substantial evidence supporting the efficacy of D2 lymphadenectomy in enhancing surgical outcomes and patient survival (4, 6). However, more extensive lymph node dissection is associated with an increased risk of postoperative complications, such as pancreatic fistula and infection, leading to prolonged hospital stays, delayed recovery, and higher healthcare costs (7–9). Postoperative pleural effusion (PPE) is a frequent complication, especially following surgery for adenocarcinoma of the esophagogastric junction (AEG) (10, 11). The unique anatomical features of AEG contribute to a higher incidence of PPE, which can significantly impair recovery. Despite this, the risk factors for PPE in AEG patients are not fully understood.

The red blood cell distribution width-to-albumin ratio (RAR) is an emerging biomarker that combines erythrocyte volume variability and serum albumin (Alb) levels, reflecting systemic inflammation and nutritional status (12, 13). It has shown significant prognostic value in various clinical contexts, including inflammatory diseases, cardiovascular disorders, and cancers, by effectively predicting mortality and severe complications (14, 15). Despite its promise, the role of RAR in predicting postoperative outcomes following gastrectomy for gastric cancer has not been thoroughly explored.

Several inflammatory and nutritional indices, such as the neutrophil-to-lymphocyte ratio (NLR), prognostic nutritional index (PNI), and the Glasgow Prognostic Score (mGPS), have been established to predict postoperative complications (16). Although these indices are useful, they often involve multiple clinical variables (such as CRP and lymphocyte count) and can be cumbersome in routine practice (17). In contrast, RAR, which is derived from two simple and routinely measured parameters—red blood cell distribution width (RDW) and Alb—offers a more accessible approach for evaluating patients’ inflammatory and nutritional status, particularly in the perioperative period. However, the comparative advantage of RAR over existing indices in predicting PPE, especially in AEG patients, has yet to be fully examined.

In this study, we performed a retrospective analysis of patients with Siewert type II/III AEG who underwent gastrectomy. Our aim was to explore the association between RAR and the development of PPE following gastrectomy in this patient group.

## 2 Materials and methods

### 2.1 Patients

**Figure 1:**
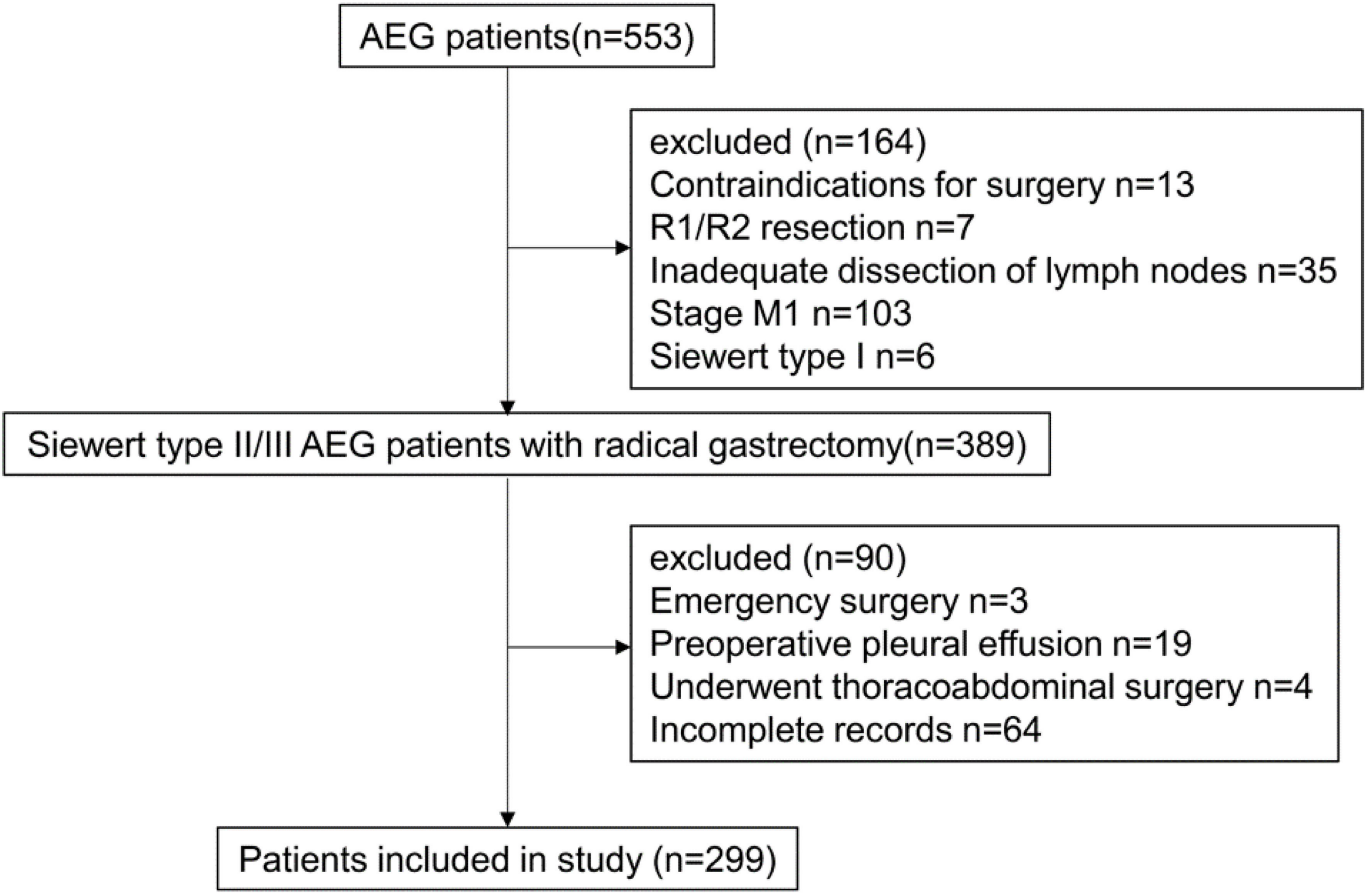
The participant flow diagram. AEG, adenocarcinoma of the esophagogastric junction.

This retrospective study encompassed patients diagnosed with Siewert type II/III AEG who underwent radical gastrectomy at the Department of General Surgery, First Affiliated Hospital of Xi’an Jiaotong University, between January 2020 and December 2024. The electronic medical records of these patients were stored in a prospectively maintained database, from which we extracted comprehensive data, encompassing baseline characteristics, clinical findings, intraoperative details, pathological information, and postoperative management details (**Figure 1**).

The study included patients who met the following criteria: (I) Histologically confirmed Siewert type II/III AEG; (II) Underwent elective radical proximal gastrectomy (PG) or total gastrectomy (TG); (III) No significant abnormalities in routine blood tests, liver and kidney function tests, and electrocardiograms, indicating that the patient is fit for surgery.

The exclusion criteria were as follows: (I) Emergency surgery due to tumor-related complications (e.g., perforation or bleeding); (II) Presence of preoperative pleural effusion; (III) History of thoracoabdominal combined surgical approach; (IV) Incomplete clinical or laboratory data.

This study has been approved by the clinical medical research ethics committee of the First Affiliated Hospital of Xi’an Jiaotong University. The study was conducted in accordance with the Declaration of Helsinki (as revised in 2013). The need for patient consent was waived due to the retrospective nature of the study.

### 2.2 Surgical procedures for gastrectomy

The same surgical team conducted either PG or TG with D1+/D2 lymph node dissection for all patients, employing either open or laparoscopic techniques in alignment with the Japanese GC treatment guidelines (18). The determination of the surgical approach (open versus laparoscopic) and the extent of lymph node dissection (D1+ versus D2) was rigorously guided by tumor stage, location, and prevailing clinical guidelines. Specifically, the selection of surgical approach and lymph node dissection adhered to the guidelines set forth by the National Comprehensive Cancer Network for gastric cancer (19) and the Japanese Gastric Cancer Treatment Guidelines (18), which offer evidence-based recommendations for gastric cancer management. The digestive tract reconstruction methods employed included Roux-en-Y esophagojejunostomy for TG and either double-tract reconstruction or esophagogastrostomy for PG. All anastomoses were executed using either circular or linear staplers, with the choice determined by the surgeons’ expertise and the specific surgical circumstances.

### 2.3 Diagnosis and treatment of postoperative pleural effusion

PPE was diagnosed through chest imaging, with any detected PPE considered a positive result. The severity of PPE was classified according to the Clavien-Dindo complication grading system, and appropriate treatment was administered to symptomatic patients (20). Initial treatment involved the administration of Alb and/or diuretics, followed by thoracic puncture for patients in whom the initial treatment was ineffective. The diagnosis of both preoperative and PPE was conducted using the same methodology.

### 2.4 Data collection

In this study, potential risk factors increasing the opportunities for PPE were included as the following: sex, age, body mass index (BMI), history of hypertension or coronary heart disease, history of chronic obstructive pulmonary disease (COPD), smoking and drinking habits, hemoglobin (Hb) level, Alb level, white blood cell (WBC) count, history of abdominal surgery, preoperative chemotherapy, T stage, N stage, lymphovascular invasion, perineural invasion, histologic type, Siewert type, Lauren type, carcinoembryonic antigen (CEA) level, carbohydrate antigen 19-9 (CA19-9) level, carbohydrate antigen 72-4 (CA72-4) level, nutritional risk, surgical method, lymph node dissection, type of gastric resection, tumor size, number of retrieved lymph nodes, number of positive lymph nodes, length of the proximal margin, status of combined organ resection, duration of operation, intraoperative blood loss, intraoperative transfusion, intraoperative Alb supplementation, and RAR. Anemia was diagnosed according to the WHO criteria: hemoglobin < 130 g/L for men and < 120 g/L for women (21). According to the NRS-2002 scores (22), patients were divided into two groups (with nutritional risk: NRS-2002 scores ≥3, without nutritional risk: NRS-2002 scores 0-2). Serum Alb < 35 g/L was defined as hypoproteinemia. Elevated WBC was defined as ≥10^9^/L.

The RAR was calculated by dividing RDW (%) by serum Alb concentration (g/dL). RDW was measured with the automatic blood analyzer (Mindray BC-6800, China), while serum Alb was assessed using the bromocresol purple method.

In this study, the initially selected of variables based on prior literature and clinical relevance, prioritizing those commonly associated with PPE in the studied population. Variables such as preoperative chemotherapy, lymphocyte count, combined organ resection, and operation time were chosen due to their established links to inflammation, nutritional status, and pulmonary complications.

Before incorporating these variables into multivariable logistic regression models, we first performed univariate logistic regression analysis to identify statistically significant factors (P < 0.05). These variables were then included in the multivariable models. In Models 2 and 3, we further included demographic, oncologic and surgical-related variables that were theoretically relevant to the outcome, such as age, sex, and tumor stage.

The selection of covariates was based on both statistical significance and biological plausibility, informed by clinical experience. For instance, lymphocyte count and operation time were included due to their known roles in inflammation and recovery, while factors like lymph node dissection and proximal margin length were considered for their impact on surgical outcomes and postoperative complications.

### 2.5 Statistical analysis

The statistical analyses in this study were conducted using SPSS version 24.0 (IBM Corp, Armonk, NY, USA) and R software (version 4.4.2, https://www.r-project.org/). Descriptive statistics and frequency tables were employed to summarize the data. Variables following a normal distribution were analyzed using Student’s t-test, while those with non-normal distributions were assessed using the Mann-Whitney U test. Categorical data were evaluated using the chi-squared test.

To avoid overparameterization, univariable logistic regression was first performed, and variables with statistical significance were entered into the multivariable models. Subsequently, stepwise models with increasing degrees of adjustment were constructed to assess the robustness of the association between RAR and PPE. The receiver operating characteristic (ROC) curve was utilized to assess the predictive capability of RAR for the risk of PPE, with the area under the curve (AUC) serving as the measure of performance. To evaluate the incremental predictive value of RAR, we compared the discrimination of a base clinical model with models additionally including RDW, Alb, or RAR. Differences in AUC were assessed using the DeLong test. The clinical utility of the model was estimated using decision curve analysis (DCA). Given the limited number of PPE events (n=85) and the number of covariates included in the fully adjusted model, we assessed the potential risk of overfitting. Internal validation was performed using bootstrap resampling (1,000 iterations) to estimate optimism-corrected AUC values.

The variable RAR was analyzed both as a continuous and a categorical variable (Quartile 1: ≤3.53, Quartile 2: 3.53–3.98, Quartile 3: 3.98–4.77, Quartile 4: >4.77) through logistic regression, with results expressed as odds ratios (OR) and 95% confidence intervals (CI). Subgroup analyses were also conducted. Statistical significance was established at P < 0.05.

This was an exploratory retrospective study, and the sample size was determined by available eligible cases during the study period.

## 3 Results

### 3.1 Patient characteristics

A total of 299 patients with Siewert type II/III AEG were enrolled in this study, 85 patients (28.43%) were diagnosed with PPE. A detailed comparison of baseline characteristics between the PPE and non-PPE groups is presented in **Table 1**. It is noteworthy that the PPE group exhibited significantly higher rates of preoperative chemotherapy, as well as reduced preoperative Hb and lymphocyte counts (P<0.05). Additionally, patients with PPE were more likely to undergo D2 lymph node dissection and combined organ resection, and they experienced longer operative times, increased intraoperative blood loss, and elevated RAR levels (P<0.05). No statistically significant differences were observed between the two groups regarding age, sex, BMI, history of hypertension or coronary heart disease, history of COPD, smoking and alcohol consumption habits, Alb levels, WBC counts, history of abdominal surgery, T stage, N stage, histologic type, Siewert classification, Lauren classification, CEA level, CA19–9 level, CA72–4 level, surgical approach, type of gastric resection, and tumor size (P>0.05).

**Table 1:** Baseline clinical characteristics of Siewert type II/III adenocarcinoma of the esophagogastric junction patients who underwent gastrectomy.

Variables	Total	Non-PPE	PPE	P
Age, years, median (IQR)	66.00 (59.00-70.00)	66.0 (59.00-70.00)	67.00 (60.00-71.00)	0.215
Sex, n (%)				0.281
Male	269(90.0%)	190(88.8%)	79(92.9%)	
Female	30(10.0%)	24(11.2%)	6(7.1%)	
BMI, kg/m2, mean ± SD	23.26 ± 3.03	23.07 ± 3.10	23.73 ± 2.80	0.107
Hypertension or coronary heart disease, n (%)				0.759
No	187(65.5%)	135(63.1%)	52(61.2%)	
Yes	112(37.5%)	79(36.9%)	33(38.8%)	
COPD, n (%)				0.717
No	290(97.0%)	208(97.2%)	82(96.5%)	
Yes	9(3.0%)	6(2.8%)	3(3.5%)	
Smoking habit, n (%)				0.457
Never smokers	182(60.9%)	126(58.9%)	56(65.9%)	
Former smokers	55(18.4%)	40(18.7%)	15(17.6%)	
Current smokers	62(20.7%)	48(22.4%)	14(16.5%)	
Drinking habit, n (%)				0.540
Never drinkers	249(83.3%)	176(82.2%)	73(85.9%)	
Former drinkers	22(7.4%)	18(8.4%)	4(4.7%)	
Current drinkers	28(9.4%)	20(9.3%)	8(9.4%)	
Hb, g/L, mean ± SD	118.50 ± 22.31	122.30 ± 20.02	108.93 ± 24.92	**0.002**
Alb, g/L, mean ± SD	35.92 ± 4.05	36.38 ± 4.07	34.74 ± 3.78	0.674
Lymphocyte, ×10^9/L, median (IQR)	1.46 (1.12-1.79)	1.52 (1.20-1.82)	1.30 (1.03-1.76)	**0.006**
WBC, ×10^12/L, median (IQR)	4.89 (4.07-5.93)	4.88 (4.12-5.82)	4.9 (3.87-6.15)	0.975
Previously laparotomy, n (%)				0.720
No	246(82.3%)	175(81.8%)	71(83.5%)	
Yes	53(17.7%)	39(18.2%)	14(16.5%)	
Preoperative chemotherapy, n (%)				**0.037**
No	222(74.2%)	166(77.6%)	56(65.9%)	
Yes	77(25.8%)	48(22.4%)	29(34.1%)	
T stage, n (%)				0.467
T0	6 (2.0%)	4 (1.9%)	2 (2.4%)	
T1	72(24.1%)	55(25.7%)	17(20.0%)	
T2	43(14.4%)	26(12.1%)	17(20.0%)	
T3	124(41.5%)	90(42.1%)	34(40.0%)	
T4	54(18.1%)	39(18.2%)	15(17.6%)	
N stage, n (%)				0.872
N0	151(50.5%)	105(49.1%)	46(54.1%)	
N1	52(17.4%)	38(17.8%)	14(16.5%)	
N2	40(13.4%)	29(13.6%)	11(12.9%)	
N3	56(18.7%)	42(19.6%)	14(16.5%)	
Histology, n (%)				0.649
Differentiated	131(43.8%)	92(43.0%)	39(45.9%)	
Undifferentiated	168(56.2%)	122(57.0%)	46(54.1%)	
Siewert type, n (%)				0.365
II	181(60.5%)	133(62.1%)	48(56.5%)	
III	118(39.5%)	81(37.9%)	37(43.5%)	
Lauren type, n (%)				0.942
Intestinal	81(27.1%)	58(27.1%)	23(27.1%)	
Diffuse	57(19.1%)	42(19.6%)	15(17.6%)	
Mixed	64(21.4%)	44(20.6%)	20(23.5%)	
Unknow	97(32.4%)	70(32.7%)	27(31.8%)	
CEA, ng/ml, median (IQR)	2.56 (1.64-4.26)	2.54 (1.54-4.25)	2.58 (1.76-4.30)	0.726
CA199, U/ml, median (IQR)	8.70 (4.96-16.18)	8.59 (5.09-16.22)	9.10 (4.74-15.12)	0.683
CA72-4, U/ml, median (IQR)	2.18 (1.30-5.26)	2.31 (1.31-5.26)	1.95 (1.26-5.25)	0.246
Operation method, n (%)				1.000
Open	12(4.0%)	9(4.2%)	3(3.5%)	
Laparoscopic	287(96.0%)	205(95.8%)	82(96.5%)	
Lymph node dissection, n (%)				**0.016**
D1+	40(13.4%)	35(16.4%)	5(5.9%)	
D2	259(86.6%)	179(83.6%)	80(94.1%)	
Type of resection, n (%)				0.146
Total gastrectomy	128(42.8%)	86(40.2%)	42(49.4%)	
Proximal gastrectomy	171(57.2%)	128(59.8%)	43(50.6%)	
Tumor size, mm, median (IQR)	3.00 (2.00-4.50)	3.00 (2.00-4.50)	3.20 (2.00-4.50)	0.772
Combined organ resection, n (%)				**<0.001**
No	290(97.0%)	213(99.5%)	77(90.6%)	
Yes	9(3.0%)	1(0.5%)	8(9.4%)	
Operation time, h, median (IQR)	4.50 (3.67-5.33)	4.00 (3.33-4.83)	5.00 (4.00-6.00)	**<0.001**
Blood loss, ml, median (IQR)	100.00 (100.00-200.00)	100.00 (60.00-200.00)	100.00 (100.00-200.00)	**0.049**
RAR, median (IQR)	3.98 (3.53-4.78)	3.85 (3.43-4.58)	4.39 (3.88-5.46)	**<0.001**

Bold values: significant P-values. PPE, postoperative pleural effusion; BMI, body mass index; COPD, chronic obstructive pulmonary disease; Hb, hemoglobin; Alb, albumin; WBC, white blood cell; CEA, carcinoembryonic antigen; CA199, carbohydrate antigen 199; CA72-4, carbohydrate antigen 72-4.

### 3.2 Association between RAR and postoperative pleural effusion

**Figure 2:**
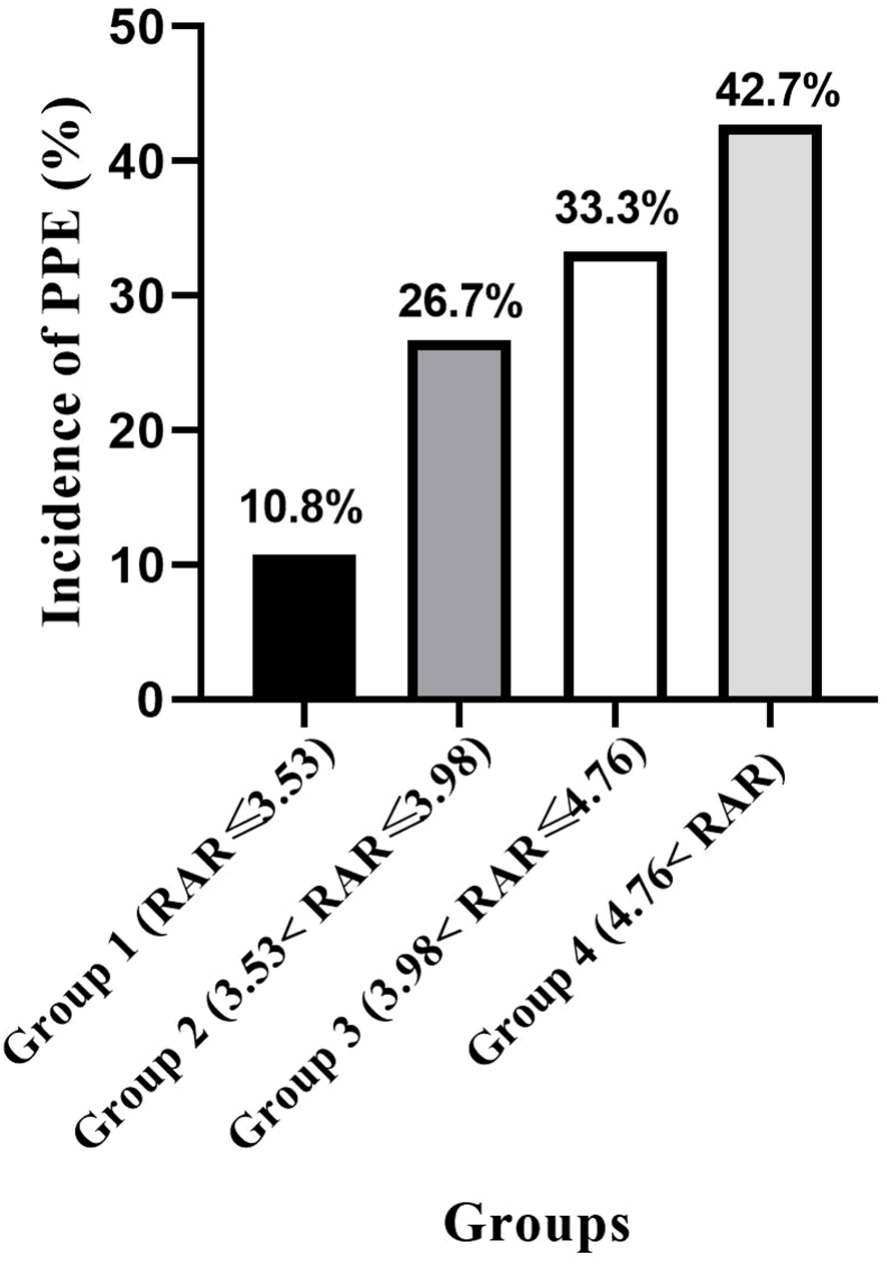
The incidence of PPE in different RAR groups. PPE, postoperative pleural effusion; RAR, red blood cell distribution width-to-albumin ratio.

Participants were further stratified into four groups according to the quartile distribution of the RAR. As depicted in **Figure 2**, there was a progressive increase in the incidence of PPE with each higher RAR quartile. Univariable and multivariable logistic regression analyses related to PPE are detailed in **Table 2**. The logistic regression analyses confirmed that an extended proximal margin (OR: 1.479, 95% CI: 1.065-2.054, P = 0.020), prolonged operation duration (OR: 1.670, 95% CI: 1.334-2.090, P<0.001), combined organ resection (OR: 13.985, 95% CI: 1.515-129.065, P = 0.020), and elevated RAR levels (OR: 1.692, 95% CI: 1.222-2.343, P = 0.002) were significant risk factors for PPE. Conversely, increased lymphocyte counts (OR: 0.475, 95% CI: 0.248-0.909, P = 0.025) remained protective factors.

**Table 2:** Univariate and multivariable analyses for postoperative pleural effusion.

Variables	Univariate			Multivariate		
	OR	95% CI	P	OR	95% CI	P
Age			0.603			
≤60	1					
>60	1.158	0.666-2.015				
Sex			0.600			
Male	1					
Female	0.601	0.237-1.527				
BMI						
≤18.5	1					
>18.5,≤25	1.397	0.376-5.193	0.618			
>25	1.702	0.438-6.624	0.443			
Hypertension or coronary heart disease			0.759			
No	1					
Yes	1.084	0.647-1.819				
COPD			0.741			
No	1					
Yes	1.268	0.310-5.191				
Smoking habit						
Never smokers	1					
Former smokers	0.844	0.4316-1.652	0.620			
Current smokers	0.656	0.335-1.287	0.220			
Drinking habit						
Never drinkers	1					
Former drinkers	0.964	0.406-2.288	P = 0.934			
Current drinkers	0.536	0.175-1.638	P = 0.274			
Hb			**<0.001**			0.438
Normal	1			1		
Reduced	2.776	1.584-4.865		1.332	0.645-2.748	
Alb			0.085			
Normal	1					
Reduced	2.230	0.895-5.558				
WBC			0.212			
Normal	1					
elevated	2.167	0.643-7.299				
Lymphocyte	0.539	0.316-0.918	**0.023**	0.475	0.248-0.909	**0.025**
Previously laparotomy			0.720			
No	1					
Yes	0.885	0.453-1.729				
Preoperative chemotherapy			**0.038**			0.790
No	1			1		
Yes	1.791	1.032-3.109		0.911	0.460-1.806	
T stage			0.676			
T0-2	1					
T3-4	0.897	0.539-1.493				
N stage			0.431			
N0	1					
N+	0.817	0.493-1352				
Lymphovascular invasion			0.493			
No	1					
Yes	0.812	0.448-1.472				
Perineural invasion			0.098			
No	1					
Yes	0.599	0.326-1.100				
Histology			0.649			
Differentiated	1					
Undifferentiated	0.889	0.537-1.474				
Siewert type			0.365			
II	1					
III	1.266	0.760-2.108				
Lauren type						
Intestinal	1					
Diffuse	0.901	0.420-1.930	0.788			
Mixed	1.146	0.560-2.345	0.709			
Unknow	0.973	0.505-1.875	0.934			
CEA	1.000	0.988-1.012	0.977			
CA199	1.001	0.999-1.003	0.274			
CA72-4	1.001	0.983-1.020	0.882			
nutritional risk			0.434			
No	1					
Yes	0.812	0.482-1.368				
Operation method			0.115			
Open	1					
Laparoscopic	1.200	0.317-4.544				
Lymph node dissection			**0.041**			0.154
D1+	1			1		
D2	2.574	1.041-6.368		2.281	0.735-7.085	
Type of resection			0.147			
Total gastrectomy	1					
Proximal gastrectomy	0.688	0.415-1.140				
Tumor size	0.992	0.882-1.116	0.894			
Retrieved lymph nodes	1.007	0.983-1.032	0.564			
Positive lymph nodes	1.003	0.959-1.049	0.909			
Length of the proximal margin	1.557	1.168-2.076	**0.003**	1.479	1.065-2.054	**0.020**
Combined organ resection			**0.004**			**0.020**
No	1			1		
Yes	22.130	2.723-179.838		13.985	1.515-129.065	
Operation time, h	1.682	1.370-2.066	**<0.001**	1.670	1.334-2.090	**<0.001**
Blood loss	1.001	1.000-1.003	0.082			
Intraoperative transfusion			**0.049**			0.512
No	1			1		
Yes	1.943	1.002-3.768		0.769	0.331-1.736	
Intraoperative albumin supplementation			0.134			
No	1					
Yes	1.661	0.855-3.225				
RAR	1.871	1.449-2.417	**<0.001**	1.692	1.222-2.343	**0.002**

Bold values: significant P-values. OR, odds ratio; CI, confidence interval; BMI, body mass index; COPD, chronic obstructive pulmonary disease; Hb, hemoglobin; Alb, albumin; WBC, white blood cell; CEA, carcinoembryonic antigen; CA199, carbohydrate antigen 199; CA72-4, carbohydrate antigen 72-4.

**Figure 3:**
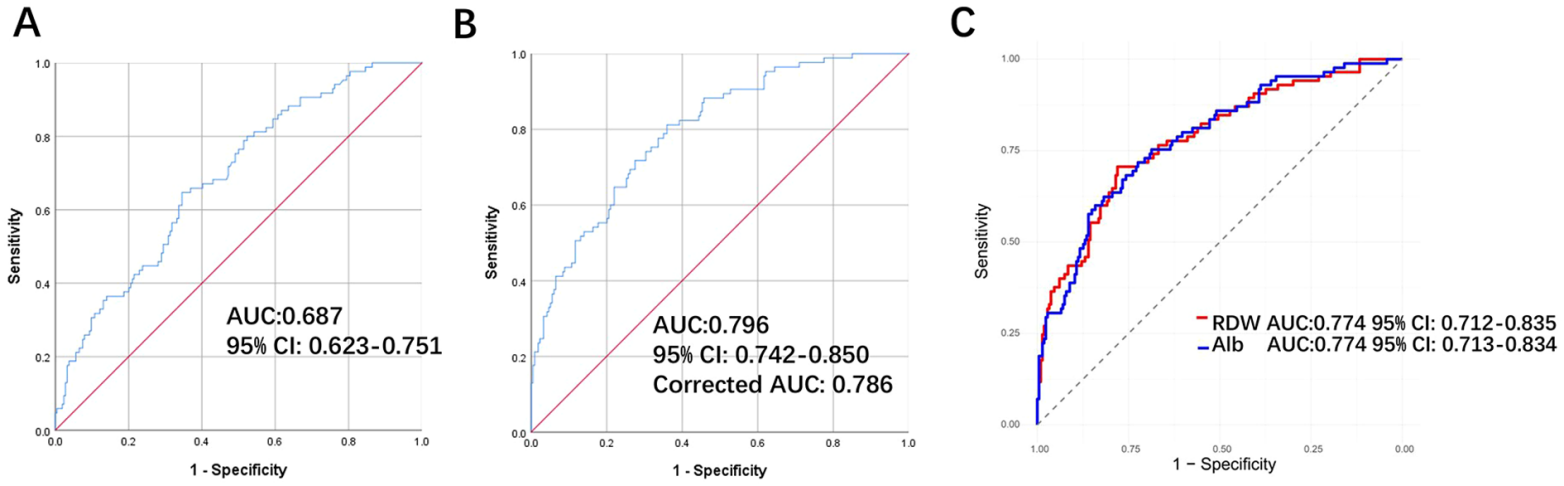
Receiver operating characteristic curve of RAR in the prediction of PPE among patients with Siewert type II/III AEG. **(A)** Crude model with no covariate adjusted. **(B)** The model was adjusted for Hb, lymphocyte counts, preoperative chemotherapy status, lymph node dissection, length of the proximal margin, combined organ resection, operation time and intraoperative transfusion. **(C)** ROC curve of the multivariable model with RDW/Alb replacing RAR. RAR, red blood cell distribution width-to-albumin ratio; PPE, postoperative pleural effusion; AEG, adenocarcinoma of the esophagogastric junction; Hb, hemoglobin; RDW, red blood cell distribution width; Alb, albumin.

**Figure 4:**
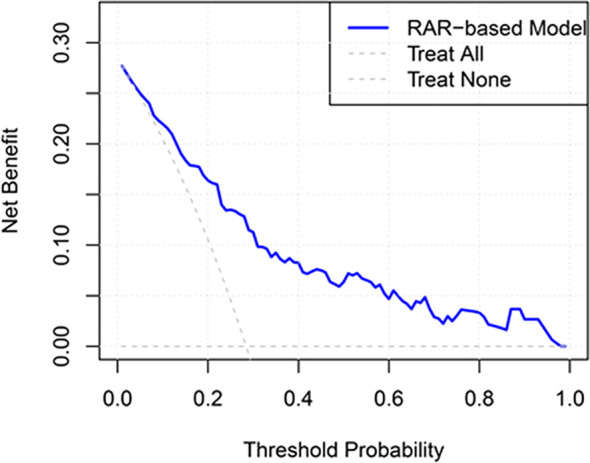
Decision curve analysis of RAR in the prediction of PPE among patients with Siewert type II/III AEG. The model was adjusted for Hb, lymphocyte counts, preoperative chemotherapy status, lymph node dissection, length of the proximal margin, combined organ resection, operation time and intraoperative transfusion. RAR, red blood cell distribution width-to-albumin ratio; PPE, postoperative pleural effusion; AEG, adenocarcinoma of the esophagogastric junction; Hb, hemoglobin.

**Table 3** illustrates a significant positive association between RAR and PPE. In the unadjusted model, participants who experienced a one-unit increase in RAR showed an 87% increase in the risk of PPE (OR = 1.871, 95% CI: 1.449–2.417, P < 0.001). This association persisted even after adjusting for relevant covariates (OR = 1.799, 95% CI: 1.274–2.541, P < 0.001). Furthermore, when RAR was categorized based on quartiles, a significant positive relationship with PPE was observed (**Table 3**). In the unadjusted model, all quartile groups demonstrated significant positive associations with PPE (P < 0.05), and the third and fourth quartiles remained significant in the adjusted models (P < 0.05). All models indicated an increased risk with higher quartiles (P for trend < 0.05). Additionally, ROC analysis was conducted to evaluate the ability of RAR to differentiate between individuals with and without PPE. **Figure 3A** shows RAR alone predicted the development of PPE in Siewert type II/III AEG patients with an AUC of 0.687 (95% CI: 0.623-0.751). After adjustment for Hb, lymphocyte counts, preoperative chemotherapy status, lymph node dissection, length of the proximal margin, combined organ resection operation time and intraoperative transfusion, the AUC was 0.796 (95% CI: 0.742-0.850) (**Figure 3B**). The AUC of RAR (0.796) was numerically higher than that of Alb and RDW alone (0.774) (**Figure 3C**), the difference did not reach statistical significance (DeLong test P > 0.05), possibly due to limited event numbers. Additionally, DCA demonstrated the favorable clinical applicability of Model 1 in predicting the incidence of PPE among Siewert type II/III AEG patients (**Figure 4**). After bootstrap internal validation, the optimism-corrected AUC was 0.786, indicating mild optimism but preserved discriminative ability.

**Table 3:** Weighted association between red blood cell distribution width-to-albumin ratio and postoperative pleural effusions among patients with Siewert type II/III adenocarcinoma of the esophagogastric junction.

Variable	Crude Model		Adjusted Model 1		Adjusted Model 2		Adjusted Model 3	
	OR (95% CI)	P	OR (95% CI)	P	OR (95% CI)	P	OR (95% CI)	P
Continuous								
Per one unit increase	1.871 (1.449-2.417)	**<0.001**	1.692 (1.222-2.343)	**0.002**	1.710 (1.226-2.385)	**0.002**	1.799 (1.274-2.541)	**0.001**
Categorical								
Quartile 1	1		1		1		1	
Quartile 2	2.946 (1.205-7.203)	**0.018**	1.967 (0.712-5.437)	0.192	1.962 (0.701-5.489)	0.199	2.168 (0.761-6.177)	0.147
Quartile 3	4.209 (1.750-10.125)	**0.001**	3.042 (1.045-8.851)	**0.041**	3.097 (1.044-9.182)	**0.042**	3.509 (1.155-10.663)	**0.027**
Quartile 4	6.140 (2.486-14.578)	**<0.001**	3.532 (1.161-10.742)	**0.026**	3.516 (1.127-10.964)	**0.030**	4.148 (1.279-13.453)	**0.018**
Ptrend		**<0.001**		**0.042**		**0.049**		**0.032**

Crude model, no covariate was adjusted; Adjusted model 1, adjusted for Hb, lymphocyte counts, preoperative chemotherapy status, lymph node dissection, length of the proximal margin, combined organ resection, operation time and intraoperative transfusion; Adjusted model 2, adjusted for age, sex, BMI, Hb, lymphocyte counts, preoperative chemotherapy status, lymph node dissection, length of the proximal margin, combined organ resection, operation time and intraoperative transfusion; Adjusted model 3, adjusted for age, sex, BMI, T stage, N stage, operation method, type of gastric resection, Hb, lymphocyte counts, preoperative chemotherapy status, lymph node dissection, length of the proximal margin combined organ resection, operation time and intraoperative transfusion. Model 1 represents a parsimonious model; Model 2 and Model 3 represents adjusted models used for sensitivity analysis. Bold values: significant P-values. OR, odds ratio; CI, confidence interval; BMI, body mass index; Hb, hemoglobin.

### 3.3 Subgroup analysis

The subgroup analysis further indicates a significant positive correlation between RAR and PPE, as presented in **Table 4**. The stratified analysis identified a significant interaction between Hb levels and RAR (P < 0.05). Notably, RAR showed a more pronounced positive association with PPE in patients without anemia compared to those with anemia. No significant differences were found in the subgroup analyses concerning age, sex, BMI, T stage, N stage, histology, Siewert type, extent of lymph node dissection, extent of gastrectomy, and RAR (P > 0.05).

**Table 4:** Stratified analyses of the weighted association between red blood cell distribution width-to-albumin ratio and postoperative pleural effusions among patients with Siewert type II/III adenocarcinoma of the esophagogastric junction.

Variable	OR (95% CI)	P	P_interaction_
Age			0.195
≤60	1.073 (0.460-2.507)	0.870	
>60	2.266 (1.383-3.711)	0.001	
Sex			0.572
Male	1.950 (1.273-2.988)	0.002	
Female	NA	NA	
BMI			0.217
≤25	1.511 (0.990-2.306)	0.06	
>25	6.13 (1.897-1.985)	0.003	
Hb			0.035
Normal	3.741 (1.418-9.868)	0.008	
Reduced	1.677 (1.093-2.572)	0.018	
T stage			0.729
T0-2	1.308 (0.641-2.667)	0.461	
T3-4	2.505 (1.424-4.406)	0.001	
N stage			0.399
N0	1.632 (0.931-2.860)	0.087	
N+	2.695 (1.394-5.212)	0.003	
Histology			0.293
Differentiated	2.780 (1.342-5.76)	0.006	
Undifferentiated	1.670 (0.959-2.908)	0.070	
Siewert type			0.506
II	1.720 (1.029-2.877)	0.039	
III	2.515 (1.250-5.058)	0.010	
Lymph node dissection			0.715
D1+	1.200 (0.283-5.093)	0.805	
D2	1.962 (1.291-2.983)	0.002	
Type of resection			0.368
Total gastrectomy	1.801 (1.152-2.816)	0.010	
Proximal gastrectomy	2.024 (1.108-3.698)	0.022	

The models were adjusted for Hb, lymphocyte counts, preoperative chemotherapy status, lymph node dissection, length of the proximal margin, combined organ resection, operation time and intraoperative transfusion, except for the corresponding stratification variable. NA, the sample size was too small for statistical evaluation. OR, odds ratio; CI, confidence interval; BMI, body mass index; Hb, hemoglobin.

## 4 Discussion

This study is the first to elucidate a significant association between the preoperative RAR and the incidence of PPE in patients with Siewert type II/III AEG. Notably, this association remained statistically significant following both univariate and multivariate regression analyses, even after adjusting for key demographic, oncologic-related, and surgical-related variables. These findings suggest that RAR could serve as a simple, clinically feasible supplementary predictive marker for PPE, potentially improving risk stratification and perioperative management in Siewert type II/III AEG surgeries. Due to the single-center and retrospective nature of this study, the clinical application of RAR requires further research and external validation.

Postoperative pulmonary complications are common, with an incidence rate of 39% (23). In patients with Siewert type II/III AEG, prior studies report the incidence of post-gastrectomy pulmonary complications to be approximately 24.7%, with PPE incidence ranging from 8.30% to 21.95% (24, 25). However, our study observed a slightly higher incidence than those reported in the literature, which may be attributed to the extensive use of laparoscopic techniques and the routine imaging examinations for all patients at our center following gastrectomy. Therefore, further research with a larger sample size is warranted to explore the incidence of PPE after gastrectomy in patients with Siewert type II/III AEG.

In prior research, RDW has been recognized as a marker of systemic inflammation and oxidative stress, linking it to a range of conditions, including cardiovascular diseases, cancer, diabetes, and chronic kidney disease (26–28). PPE, a frequent pulmonary complication following GC surgery, is also associated with inflammatory states. The development of PPE involves multiple pathophysiological mechanisms, including increased capillary permeability, leading to fluid and protein leakage into the interstitial space; impaired lymphatic drainage, causing fluid accumulation in the pleural cavity; and low colloid osmotic pressure, often resulting from hypoalbuminemia, which exacerbates fluid transudation (29–31). Alb supplementation plays a fundamental role in managing complications related to hypoalbuminemia, highlighting the importance of maintaining a balance between inflammation and nutrition in clinical outcomes (31). The RAR combines these two biomarkers, providing a composite measure that reflects both inflammatory activity and nutritional status (32, 33). Due to its integrated nature, RAR has the potential to predict postoperative complications, including PPE, by reflecting the underlying pathophysiological processes that drive fluid accumulation in the pleural space. AS reported by Masahiro et al. (34), Zhijian et al. (35), as well as Yuki et al. (36), inflammatory and albumin-related biomarkers, such as the PNI and high C-reactive Alb ratio, are associated with the occurrence of short-term complications after GC surgery. In the present study, the discriminative ability of RAR ranged from 0.687 to 0.796 depending on model adjustment, indicating moderate predictive performance. These values are broadly comparable to those reported for other inflammatory or nutritional indices in gastric or upper gastrointestinal surgery (37, 38), rather than clearly superior. However, compared to existing inflammatory or nutritional scores, RAR offers a more simplified approach that may enable rapid risk stratification without additional laboratory burden. Our findings reveal a significant and independent association between elevated RAR and the occurrence of PPE, indicating that RAR may serve as pragmatic, clinically feasible supplementary predictive tool for identifying patients at elevated risk of this postoperative complication. Nevertheless, whether it provides superior predictive value over established indices warrants further comparative studies.

Moreover, the robustness of the findings was corroborated through multivariable adjustment and subgroup analyses. The multivariable ROC analysis produced an AUC of 0.796, signifying good predictive performance. Although the improvement in AUC was modest and did not reach statistical significance, the consistent direction of effect and the biological rationale supporting the combined marker suggest that RAR may offer incremental information. However, this result should be interpreted cautiously and requires validation in larger cohorts. Subgroup analysis demonstrated that most subgroups did not exhibit significant differences, reinforcing the robustness of the results. This indicates a clear association between RAR and the incidence of PPE, with no varying trends across different subgroup populations. The sole exception was observed between the anemia and non-anemia groups, where a significant interaction (Pinteraction) was detected. Nevertheless, in both the anemia and non-anemia groups, each unit increase in RAR was associated with an OR greater than 1. This suggests that although the magnitude of increased PPE risk associated with elevated RAR may vary across different Hb levels, the consistent finding is that higher RAR corresponds to an elevated risk of PPE in both groups.

This study is subject to several limitations. Firstly, it was conducted as a single-center, nonrandomized, retrospective analysis, which may introduce selection and sampling biases. Secondly, despite adjustments for various confounders, we acknowledge the potential risk of residual confounding due to unavailable variables, such as other inflammatory biomarkers, pulmonary specific risk factors, fluid balance, comorbidity scores and certain intraoperative variables. Additionally, surgeon variability in surgical techniques, such as the choice between open and laparoscopic procedures, and differences in early postoperative management strategies, including fluid and nutritional support, could also influence postoperative outcomes like pleural effusion. Conversely, to minimize over-adjustment, variables considered potential mediators on the causal pathway between inflammation/malnutrition and PPE were not indiscriminately included. Thirdly, data on overall survival and other long-term outcomes were not collected in our study. Therefore, the relationship between the RAR and long-term prognosis in patients with Siewert type II/III AEG warrants further investigation. Lastly, due to the relatively limited number of events and the number of candidate predictors, overfitting cannot be fully excluded. Although internal validation was performed, the reported AUC may still represent optimistic performance and should be confirmed in larger external cohorts. We are actively planning a multi-center validation study soon to evaluate RAR’s robustness in diverse patient groups.

## 5 Conclusion

This study demonstrates a significant association between elevated RAR levels and clinically relevant PPE in patients with Siewert type II/III AEG, underscoring the impact of systemic inflammation and malnutrition on postoperative complications. RAR may serve as a pragmatic, clinically feasible supplementary index for PPE, facilitating early detection and personalized treatment strategies. However, due to the study’s single-center, retrospective design and absence of external validation, further research with external cohorts is needed before RAR can be considered for widespread clinical implementation.

## Data Availability

The original contributions presented in the study are included in the article/supplementary material. Further inquiries can be directed to the corresponding author.
